# Evaluation of the Anti-Tumor Activity of the Humanized Monoclonal Antibody NEO-201 in Preclinical Models of Ovarian Cancer

**DOI:** 10.3389/fonc.2020.00805

**Published:** 2020-06-19

**Authors:** Kristen P. Zeligs, Maria Pia Morelli, Justin M. David, Monica Neuman, Lidia Hernandez, Stephen Hewitt, Michelle Ozaki, Akosua Osei-Tutu, David Anderson, Thorkell Andresson, Sudipto Das, Justin Lack, Abdalla Abdelmaksoud, Massimo Fantini, Philip M. Arlen, Kwong Y. Tsang, Christina M. Annunziata

**Affiliations:** ^1^Women's Malignancy Branch, Center for Cancer Research, National Cancer Institute, National Institutes of Health, Bethesda, MD, United States; ^2^Precision Biologics, Inc., Bethesda, MD, United States; ^3^Protein Characterization Laboratory of the Cancer Research Program (CRTP)/Mass Spectrometry Center, National Institutes of Health, Fredrick, MD, United States; ^4^NIAID Collaborative Bioinformatics Resource (NCBR), National Institute of Allergy and Infectious Diseases, National Institutes of Health, Bethesda, MD, United States; ^5^Frederick National Laboratory for Cancer Research, Advanced Biomedical Computational Science, Fredrick, MD, United States; ^6^Frederick National Laboratory for Cancer Research, CCR Collaborative Bioinformatics Resource, Fredrick, MD, United States

**Keywords:** monoclonal antibody, tumor-associated antigen, antibody-dependent cellular cytotoxicity, natural killer cell, carcinoembryonic antigen-related cell adhesion molecule 5 (CEACAM5), carcinoembryonic antigen-related cell adhesion molecule (CEACAM6)

## Abstract

**Purpose:** Despite high initial response rates with cytoreductive surgery, conventional chemotherapy and the incorporation of biologic agents, ovarian cancer patients often relapse and die from their disease. New approaches are needed to improve patient outcomes. This study was designed to evaluate the antitumor activity of NEO-201 monoclonal antibody (mAb) in preclinical models of ovarian cancer where the NEO-201 target is highly expressed.

**Experimental Design:** Functional analysis of NEO-201 against tumor cell lines was performed by antibody-dependent cellular cytotoxicity (ADCC) assays. Binding of NEO-201 to tumor tissues and cell lines were determined by immunohistochemistry (IHC) and flow cytometry, respectively. Further characterization of the antigen recognized by NEO-201 was performed by mass spectrometry. Ovarian cancer models were used to evaluate the anti-tumor activity of NEO-201 *in vivo*. NEO-201 at a concentration of 250 g/mouse was injected intraperitoneally (IP) on days 1, 4, and 8. Human PBMCs were injected IP simultaneously as effector cells.

**Results:** Both IHC and flow cytometry revealed that NEO-201 binds prominently to the colon, pancreatic, and mucinous ovarian cancer tissues and cell lines. Immunoprecipitation of the antigen recognized by NEO-201 was performed in human ovarian, colon, and pancreatic cancer cell lines. From these screening, carcinoembryonic antigen-related cell adhesion molecule 5 (CEACAM5) and CEACAM6 were identified as the most likely targets of NEO-201. Our results confirmed that NEO-201 binds different types of cancers; the binding is highly selective for the tumor cells without cross reactivity with the surrounding healthy tissue. Functional analysis revealed that NEO-201 mediates ADCC killing against human ovarian and colorectal carcinoma cell lines *in vitro*. In addition, NEO-201 inhibited tumor growth in the presence of activated human PBMCs in orthotopic mouse models of both primary and metastatic ovarian cancer. Importantly, NEO-201 prolonged survival of tumor-bearing mice.

**Conclusions:** These data suggested that NEO-201 has an antitumor activity against tumor cells expressing its antigen. Targeting an antigen expressed in tumors, but not in normal tissues, allows patient selection for optimal treatment. These findings strongly indicate that NEO-201 warrants clinical testing as both a novel therapeutic and diagnostic agent for treatment of ovarian carcinomas. A first in human clinical trial evaluating NEO-201 in adults with chemo-resistant solid tumors is ongoing at the NIH clinical Center.

## Introduction

Ovarian cancer is the most lethal gynecologic malignancy in the United States. Although it accounts for only 3% of cancers among women, it is the fifth most common cause of cancer-related death (1). Most patients are diagnosed when distant metastatic spread is already present. To date, the treatment of primary and recurrent ovarian cancer groups most epithelial ovarian subtypes together, in a common therapeutic approach. Primary mucinous carcinoma of the ovary represents a small subset of epithelial ovarian carcinoma and is histologically, molecularly, and clinically distinct from the other subtypes.

Over the last 10 years, incorporation of precision therapy and immunotherapy has led to important paradigm changes for the treatment of cancer patients. Cancer immunotherapy is aimed to enhance the power of the host immune system for the treatment of malignancy. Recent efforts in cancer therapeutics have focused on the development of immune checkpoint inhibitors, which are FDA approved for the treatment of certain tumor types. To date, this class of drugs in ovarian cancer has shown limited activity when used as monotherapy (2). Ongoing trials are evaluating the activity of PD-1/PD-L1 inhibition in combinations with other therapeutic agents that have shown activity in ovarian cancers such as VEGF and PARP inhibitors (3).

The majority of recent developments in immunotherapy strategies, including immune checkpoint inhibitors (4), vaccines (5), and engineered chimeric T-cell receptors (6), have focused on boosting the adaptive immune system. Additionally, the innate immune response can play an important antitumor role with direct tumoricidal activity, and/or indirect activity through the processing and presenting of tumor antigens to T cells (7). It is well known that monoclonal antibodies (mAbs), such as rituximab and trastuzumab, can mediate antibody-dependent cellular cytotoxicity (ADCC) (8). Natural killer (NK), neutrophils, and other myeloid cells can also kill through ADCC, a process by which engagement of FcγRs results in the release of cytotoxic granules (8). In addition to ADCC, opsonization of tumor cells with antitumor mAbs can lead to macrophages' antibody-dependent phagocytosis (ADPC) and to complement-dependent cytotoxicity (CDC) (9).

NEO-201 is a humanized IgG1 monoclonal antibody (mAb) derived from an immunogenic cancer vaccine. NEO-201 was selected for its tumor specificity and its association with clinical response. It was generated using the Hollinshead allogenic colorectal cancer vaccine platform (10), where tumor-associated antigens (TAA), derived from tumor membrane fractions pooled from colorectal cancer surgical specimens, were screened for delayed-type hypersensitivity and evaluated in clinical trials (11). Those patients who developed a sustained IgG response and a cell-mediated response against the vaccine achieved significant anti-tumor response and increased overall survival (12). NEO-201 binds specifically to a wide range of human cancer cells and tumor tissues, but not with healthy normal tissues. NEO-201 showed to have both ADCC and CDC activity against cancer cell lines *in vitro* (10, 13) and to counteract the growth of human pancreatic xenograft tumors *in vivo* (10). In the present work, we sought to further characterize the antigen recognized by NEO-201, and to demonstrate its efficacy in preclinical ovarian models. We performed mass spectrometry analysis to identify its target antigen. Exome sequencing was conducted to identify mutations shared by cell lines expressing the antigen recognized by NEO-201 and to identify possible effector pathways.

## Materials and Methods

### Drug

NEO-201 humanized monoclonal antibody was generated and provided by Precision Biologics, Bethesda, MD, USA (10).

### Cell Lines and Culture

The following human colorectal (CRC), ovarian (OV) and pancreatic (PDAC) cancer cell lines were obtained from the American Type Culture Collection (ATCC) or National Cancer Institute (NCI)-60: LS174T (CRC), SW480 (CRC), Ovcar8 (OV), Ovacar5 (OV), PEO1 (OV), PEO4 (OV), PEO5 (OV), OV90 (OV), ASPC-1 (PDAC), BxPC3 (PDAC), CFPAC-1 (PDAC). Cells were grown in RPMI medium (Corning Life Science, Manassas, VA, USA) supplemented with 10% USA-sourced and heat-inactivated HyClone Fetal Bovine Serum (FBS; GE Healthcare Life Sciences, Issaquah, WA, USA), 1% penicillin/streptomycin (Corning Life Science, Manassas, VA, USA) and maintained at 37°C in incubator under 5% CO_2_. Cell lines were authenticated via short tandem repeat at the Frederick National Laboratory.

The highly active natural killer (haNK) cell line was obtained from Nantkwest and cultured with X-VIVO media (Lonza, Basilea, Switzerland) enriched with L-glutamine and 5% heat-inactivated human AB serum (Gemini Bio-Products, West Sacramento, CA, USA) as previously described (14). Cells used for tumor induction were tested by Molecular Testing of Biological Materials (MTBM) as required by the NCI ACUC Committee and confirmed to contain no mouse viruses. Human peripheral blood mononuclear cells (PBMCs) were collected from anonymous healthy donors under protocol 99-CC-0168, approved by the Institutional Review Board of the National Cancer Institute.

### Immunoblotting

Cells were seeded in 6-well plates and allowed to grow for 24 h. Protein lysates were prepared in radioimmunoprecipitation assay (RIPA) buffer (Santa Cruz Biotechnology Inc, Dallas, TX, USA) according to manufacturer's protocol. The total protein was determined using the BCA Protein Assay Kit (Thermo Fisher Scientific, Waltham, MA, USA). Twenty-five micrograms of total protein were loaded onto a 4–12% gradient gel, electrophoresed, and transferred to nitrocellulose using the NuPage system (Invitrogen, Thermo Fisher Scientific, Waltham, MA, USA). Membranes were blocked for 1 h in 5% Milk in TBS-Tween blocking buffer and incubated overnight with NEO-201 (1 μg/ml) at 4°C. Following incubation with NEO-201, membranes were washed three times for 10 min in TBS-Tween and then incubated with the appropriate secondary goat anti-human IgG1 Fc-HPR (1:10,000). Membranes were stripped and probed with GAPDH (1:10,000) loading control. Blots were developed using Supersignal Chemiluminescent Substrate system (Thermo Fisher Scientific, Waltham, MA, USA). Immunoblot experiments were done in triplicate.

### Immunohistochemistry (IHC)

Formalin-fixed, paraffin-embedded (FFPE) sections of human tumor samples and non-malignant controls were analyzed for NEO-201 target protein expression using immunohistochemistry. Staining was performed manually. Antibody specifications and staining conditions were optimized on control whole colon cancer tissue samples, and negative controls consisted of sections that underwent similar staining procedures with an IgG control antibody of the corresponding isotype. Tissue microarray analysis was performed on 21 colon cancer, 24 lung cancer, 19 breast cancer, 11 lymphoma, 11 melanoma, and 7 glioblastoma multiforme. Tissue microarray of 627 ovarian tumor samples was obtained from Roswell Park Cancer Institute and contained tumor tissues from different subtypes of ovarian cancer, including 446 serous adenocarcinomas, 37 germinal cell tumor, 26 clear cell, 23 endometroid, 22 adenocarcinomas NOS, 22 mucinous adenocarcinoma, 18 sarcomas, 9 transitional cell, 9 carcinoma, 2 signet cell carcinoma, and 13 “other” subtype. Tissues were scored for the expression of the antigen recognized by NEO-201 and percentage of positive tumor tissue. A score of 2+ was given to those tumor tissues with a complete staining of the membrane in more than 10% of the sample analyzed and a score of 1+ to those tumor tissues with a complete staining of the membrane in <10% of the tissue analyzed.

### Flow Cytometry

Expression of tumor antigens on tumor cells was analyzed by flow cytometry. Tumor cells (1.0 × 10^6^) were harvested and first incubated with 1 μl per test of LIVE/DEAD Fixable Aqua (Thermo Fisher Scientific, Waltham, MA, USA) in 1× phosphate-buffered saline (PBS) for 30 min at 4°C to accomplish live vs. dead cell discrimination. Cells were then centrifuged, washed twice with cold PBS, and then stained in 1× PBS + 1% BSA (Teknova, Hollister, CA, USA) for 30 min at 4°C with the following anti-human mAbs: Pacific Blue-conjugated or PE-conjugated NEO-201 antibody (BioLegend, San Diego, CA, USA), CEACAM5-FITC (clone C365D3), CEACAM6-PE (clone KOR-SA3544; ThermoFisher Scientific, Waltham, MA, USA). After staining, cells were washed twice with cold PBS and examined using a FACSVerse flow cytometer (BD Biosciences, San Jose, CA, USA). Analysis of cellular fluorescence was performed using BD FACSuite software (BD Biosciences, San Jose, CA, USA). Positivity was determined using fluorescence-minus-one controls.

### Proliferation Assay

Antiproliferative effects of NEO-201 were determined using sodium 3,3′-[1(phenylamino)carbonyl]-3,4-tetrazolium]-3is(4-methoxy-6-nitro) benzene sulfonic acid hydrate (XTT) assay as previously described (15). Briefly, cells in logarithmic growth phase were transferred to 96-well flat-bottomed plates with lids. Cell suspensions containing 5 × 10^3^ cells/well were plated and incubated overnight and then treated with different concentrations of NEO-201 for 72 h. After treatment, cell viability was assessed by incubating cultures with 25 μl of XTT freshly mixed with PMS (Sigma), and absorbances were read at a measured timepoint using a Tecan plate reader (Research Triangle Park, NC, USA) as previously described. IC50 was calculated using CompuSyn software. The median dose was obtained from the anti-log of the x-intercept of the median effect plot: log (Fa/Fu) = m^*^log (D) – m^*^log (Dm) where Fa is the Fraction affected, Fu is the Fraction unaffected, and m is the slope.

### Antibody-Dependent Cellular Cytotoxicity (ADCC) Assay

To evaluate the ADCC activity of NEO-201 against human carcinoma cell lines, both radioactive and non-radioactive ADCC assays were performed. Non-radioactive ADCC assay was performed using a previously described procedure (10) using human cancer cell lines as target cells. Natural killer (NK) cells from normal donor and irradiated haNKs (10 Gy) were used as effector cells. For non-radioactive ADCC assay, target cells were labeled with 10 μM calcein AM cell-permanent dye, for 30 min and then seeded in triplicate at 3.0 × 10^3^ cells/well into black-walled flat-bottomed 96-well plates. Then, human IgG1 isotype control antibody (Thermo Fisher Scientific, Waltham, MA, USA) or NEO-201 antibody was added to target cells at different concentrations. haNK cells were simultaneously added at specific effector-to-target (E:T) ratios. After 4 h of incubation at 37°C, 1.67 μg/ml of propidium iodide (PI; Thermo Fisher Scientific, Waltham, MA, USA) was added to each well, the plate was imaged using the Celigo Imaging Cytometer (Nexcelom Bioscence LLC, Lawrence, MA, USA), and the numbers of live target cells (calcein AM+/PI–) vs. dead cells (calcein AM+/PI+ or calcein AM–/PI+) were analyzed and recorded by the Celigo Imaging Cytometer analysis software.

For radioactive ADCC assay, chromium release assays were performed using NK cells from human healthy donors. Briefly, NK cells were obtained by negative selection from human healthy donor PBMCs using the EasySep Human NK Cell Isolation Kit (StemCell Technologies, Vancouver, BC, Canada) according to the manufacturer's protocol. Purified NK cells were incubated overnight in RPMI-1640 medium supplemented with L-glutamine, 10% FBS, and antibiotics prior to be used as effector cells in the assay. On the day of the assay, cancer cells were labeled with ^51^Chromium and then used as target cells in presence of 1 μg/ml of human IgG1 isotype control antibody or NEO-201 antibody at different concentrations. NK cells were added simultaneously at specific effector-to-target (E:T) ratios. Specific lysis was calculated as % specific lysis = 100 – [(average live target cell count for antibody treated samples/average live target count for control samples) Å~100].

### ELISA

Ninety-six-well plates were first coated overnight at 4°C with 100 μl/well of 400 ng/ml recombinant human CEACAM1, 5, 6, and 8 protein (Acro Biosciences) diluted in 0.2 M sodium carbonate–bicarbonate buffer pH 9.4. Plates were washed with 1× Tris-buffered saline (TBS) + 0.05% Tween-20 and then blocked with 200 μl/well of 5% milk in 1× TBS for 1 h at 37°C. Plates were washed, and then 100 μl/well of NEO-201 antibody was added in two-fold serial dilution from 20 ng/ml to 0.156 ng/ml and incubated for 1 h at 37°C. Plates were washed, and 100 μl/well of donkey anti-human IgG antibody peroxidase conjugate (VWR) at a 1:10,000 dilution was added to the plate and incubated for 1 h at 37°C. Plates were washed, and 100 μl/well of tetramethylbenzidine (TMB) substrate solution (VWR) was added and incubated for 10 min at RT in the dark. The reaction was stopped by adding 50 μl/well of 2 N H_2_SO_4_, and absorption at 450 nm was read using a FLUOstar Omega plate reader (BMG Labtech).

### Mass Spectrometry

NEO-201 target antigen identification was performed by mass spectrometry. Briefly, 100 μg of total protein extracted from OV90, CFPAC1, OVCAR8 human cell lines, and protein A beads were incubated with 1 μg of NEO-201 and immunoprecipitated. A dose titration was performed to identify an optimal dose of NEO-201 to immunoprecipitate the proteins for the mass spectrometry analysis. One microgram and 10 ng of NEO-201 were used in the analysis. To identify the proteins bound by NEO-201, those proteins that were common in both OV90 and CFPAC1 were considered as potential targets, and those proteins identified also by the beads and the OVCAR8 were considered as non-specific binding and subtracted from the analysis. PSMs indicate the number of peptides identified of each of those proteins, and the more the number of peptides identified, the more the confidence is in the data.

### Plasmid Overexpression and Immunoblot

Overexpression experiments were performed in epithelial human embryonic kidney cell line HEK293T. Expression vectors with the incorporated CEACAM5 or CEACAM6 complementary DNAs were generated using a DHFR mammalian expression vector as the DNA of each plasmid (or empty original vector) was transiently transfected using Lipofectamine 2000 reagent (Invitrogen) into 1 × 10^6^ HEK293T cells (80 to 90% confluent) and were seeded on a 6-well plate and cultured for an additional 48–72 h. Then, the cells were harvested and lysed. Whole cell lysates and molecular weight marker standards were applied (50 μg/lane) to polyacrylamide gel and run through electrophoresis, transferred on a nitrocellulose membrane, and subjected to Western blot analysis. Primary antibodies were mouse anti-human CEACAM5 clone CB30 (Cell Signaling Technology, Danvers, MA, USA), mouse anti-human CEACAM6 clone 9A6 (Abcam, Cambridge, UK), and NEO-201.

### RNA Interference

Cells were seeded into 6-well plates and transfected with 100 nM of Dharmacon ON-TARGETplus SMARTpool siRNAs specific for CEACAM5, CEACAM6, or a non-targeting control (Horizon Discovery Group, Cambridge, UK) using 4 μl of DharmaFECT 2 transfection reagent (Horizon Discovery Group, Cambridge, UK) per transfection according to the manufacturer's instructions. Cells were incubated for at least 72 h prior to use.

### Mutational Analysis

DNA was extracted using the DNAasy Plus mini kit (Qiagen, Valencia, CA, USA) according to the manufacturer's protocol. For whole exome sequencing (WES), DNA libraries were prepared using Agilent SureSelectXT Human All Exon V5 plus UTR target enrichment kit, and samples were pooled and sequenced on an Illumina HiSeq2500 with TruSeq V4 chemistry. Alignment and variant calling was performed using the CCBR Pipeliner (https://github.com/CCBR/Pipeliner) tool on NIH's Biowulf cluster. Reads were trimmed using Trimmomatic v0.33 (16) and mapped to the hs37d5 version of the human reference genome (ftp://ftp.1000genomes.ebi.ac.uk/vol1/ftp/technical/reference/phase2_reference_assembly_sequence/hs37d5.fa.gz) using BWA-mem v07.15. BAM files were processed using Samtools v1.3 (http://www.htslib.org/) (17), and duplicates were marked using Picard v2.1.1 (http://broadinstitute.github.io/picard/). GATK v3.5.0 (18) was used to perform indel realignment and base recalibration. Read- and alignment-level quality control visualization was performed using MultiQC v1.1 (http://multiqc.info/) to aggregate QC metrics from FastQC (http://www.bioinformatics.babraham.ac.uk/projects/fastqc/), FastQ Screen (https://www.bioinformatics.babraham.ac.uk/projects/fastq_screen/), Picard, bamtools (19), stats (http://github.org/pezmaster31/bamtools), and trimmomatic. Variant calling was performed with MuTect2 (20). A panel of normals, developed from ExAC r0.3.1 (21) and the 1,000 Genomes Project (22), including only variants >0.001 in frequency in the general population was used in cases without a matched germline. Somatic variants with an allele frequency of <0.05 were excluded. Variants were annotated using Oncotator v1.9.1.0 (23) (http://portals.broadinstitute.org/oncotator/).

### Assessment of NEO-201 Activity on Tumor Growth and Survival *in vivo*

#### Primary Ovarian Cancer Model

Female athymic nude mice, 6–8 weeks old, were maintained on a 12-h light/dark cycle, with food and water provided *ad libitum*. Briefly, 2.5 × 10^5^ OV90 cells were injected into the right ovarian bursa, and 5 μl of PBS was injected into the contralateral ovarian bursa. When tumors reached an average of 100–300 mm^3^ of volume, mice were randomized into four treatment groups. Animals received treatment with either PBS/IgG as vehicle control, activated PBMCs with IgG, NEO-201 250 μg/mouse, or activated PBMCs with NEO-201. NEO-201 was administered at a dose of 250 μg/mouse IP on days 1, 4, and 8 of treatment, PBS/IgG-control was administered on the same days at a dose of 250 μg/mouse. Before injection, PBMCs were cultured overnight in RPMI media supplemented with IL-2 at 200 U/ml. A total of 500 μl with 8 × 10^6^ PBMCs was inoculated by intraperitoneal (IP) injection into each mouse on days 2, 5, and 9 of treatment. Mice were followed for signs of toxicity, and body weight was measured three times a week. Orthotopic tumor growth was assessed by ultrasound once weekly, and tumor volumes were calculated according to the formula of volume = (length × width^2^)/0.52. Mice were euthanized 2 weeks after treatment completion.

#### Metastatic Ovarian Cancer Model

To assess the effect of NEO-201 on survival, 1 × 10^6^ OV90 cells were injected into the peritoneal cavity of each mouse. Tumors were allowed to grow for 2 weeks before mice were randomized into one of the four treatment groups described above. Mice were evaluated biweekly for signs of drug-related toxicity and disease progression based on distress, physical exam changes, and cachexia. Animal care was provided in accordance with the procedures in the Guide for the Care and Use of Laboratory Animals. Experiments were carried out according to a protocol approved by the NCI Animal Care and Use Committee.

### Ultrasound Imaging

Mice were anesthetized with isoflurane via nose cone and placed dorsum up on moveable platform with arms and legs taped to the platform. The Vevo-2100 system with a 3D motor and 40-MHz probe was utilized. The platform was angled away from the investigated side. Ultrasound gel was placed over the lateral lumbar area and the motor-operated probe oriented transversely over area. The kidney was located with the ipsilateral ovary often localized at the inferior pole of the kidney, when no xenograft was present. Presence of ovarian artery and vein were confirmed by color Doppler ultrasound. A three-dimensional (3D) image was acquired by computerized 2D images obtained every 50 μm along the axis. Ovaries and ovarian tumor xenografts were analyzed for 3D volume measurement in open mode on Vevo Lab 2.1.0 software.

### Statistical Analysis

NEO-201 induced ADCC activity in “*in vitro*” model was evaluated by ordinary one-way ANOVA. Significant differences between the different mice treatment groups were evaluated by Kruskal–Wallis test, using GraphPad Prism 7.0 software (GraphPad Software, La Jolla, CA, USA). Significant differences in survival between the treatment groups were evaluated by Mantel–Cox, using GraphPad Prism 7.0 software. Differences were considered significant when the value of *p* < 0.05.

## Results

### Expression Profile of NEO-201 Binding in Patient Tumor Tissues and Human Cancer Cell Lines

**Figure 1:**
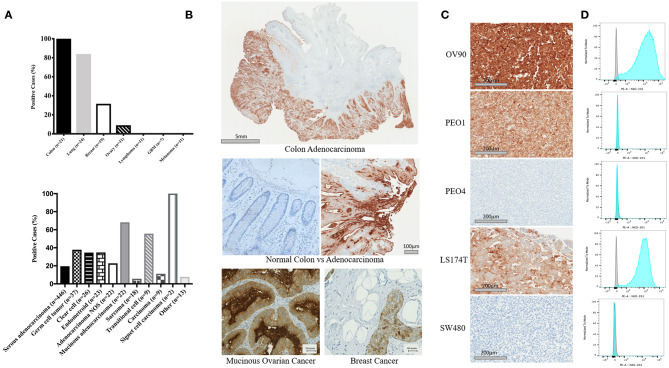
Expression profile of the antigen recognized by NEO-201 in patient tumor tissues and human cancer cell lines. **(A,B)** NEO-201 binding in colon (*n* = 21), lung (*n* = 24), breast (*n* = 19), and ovarian (*n* = 11) cancers, lymphoma (*n* = 11), glioblastoma multiforme (*n* = 7), and melanoma (*n* = 11) was measured by immunohistochemistry (IHC). Additionally, the degree of expression of the antigen recognized by NEO-201 in 627 tissues from more than 10 different ovarian cancer subtypes including serous, germ cell, clear cell, endometroid, mucinous, sarcoma, transitional, and adenocarcinoma NOS, was evaluated by tissue microarray analysis (TMA). Tissues were scored for positive vs. negative expression of the antigen recognized by NEO-201 and for percentage of positive tumor tissue. Those tissues with a complete staining of the membrane in more than 10% of the samples analyzed were given a 2+ score, while those with a complete staining of the membrane in <10% of the tissue analyzed were given a 1+. **(C)** Cell pellet from the ovarian cancer cell lines OV90, PEO1, PEO4, and colorectal cancer cell lines SW480 and LS174T were screened for NEO-201 binding by IHC. **(D)** NEO-201 binding on cancer cell line model was confirmed by FACS analysis. Cells were incubated with NEO-201 PE-conjugated antibody and then analyzed.

NEO-201 binding was evaluated by immunohistochemistry (IHC) in patient tissues from 21 colon, 24 lung, 19 breast, and 11 ovarian cancers. Also, we tested the NEO-201 binding in 11 tissues from lymphoma and melanoma, and seven glioblastomas. Respective normal tissues were tested as well (Figures 1A,B). All of the tissues from colon cancer patients resulted positive for NEO-201 staining, 84% (20/24) of the lung, 31.6% (6/19) of the breast, and 9% (1/11) of the ovarian cancer patient tissues were positive, while the respective healthy tissues surrounding the tumor were negative for NEO-201 binding. Additionally, no stain was detected in tissues from patients with lymphoma, glioblastoma, and melanoma. We further assessed the degree of NEO-201 binding in the tissues from patients with different ovarian cancer subtypes, using a tissue microarray (TMA) containing 627 ovarian cancer samples including 11 ovarian cancer histological subtype (Figure 1A, bottom). Interestingly, mucinous adenocarcinoma showed the highest percentage of positive samples among all the histological subtypes analyzed, with 68.2% positive for NEO-201 staining, and 59% (13/22) had a 2+ score. Serous adenocarcinoma and germinal cell tumors showed 20% (87/446) and 38% (14/37) positive staining, respectively. In order to identify cell line models representative of the human samples, we created a cell pellet array of ovarian and colon cancer cell lines and probed them in the same manner as the patient tissue microarrays (Figure 1C). IHC results were verified by flow cytometry except in PEO1 in which staining was discrepant (Figure 1D). Ovarian cancer cell line OV90 and colon cancer cell line LS174T showed strong staining with both techniques.

### NEO-201 Binds to Carcinoembryonic Antigen-Related Cell Adhesion Molecule (CEACAM) 5 and 6

**Figure 2:**
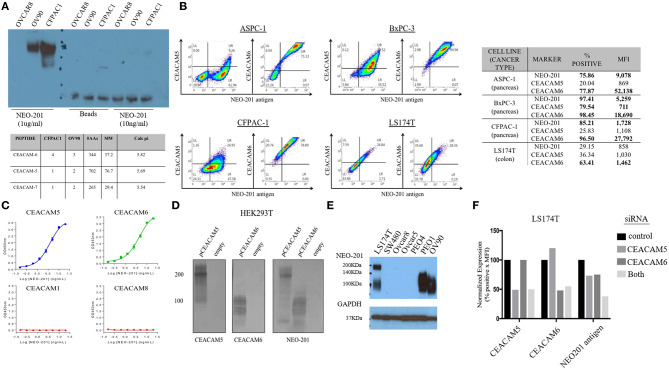
NEO-201 binds to CEACAM 5 and 6. **(A)** To identify the antigen recognized by NEO-201, 100 μg of proteins extracted from OV90, CFPAC1, and OVCAR8 was initially immunoprecipitated with 1 μg, 10 ng of NEO-201 and protein A beads. One microgram of NEO-201 showed the best results in terms of protein isolation and was used to perform intraperitoneal (IP) injection for the protein sample that was then analyzed by mass spectrometry. Peptide suggesting the glycosylated form of CEACAM-5 and−6 was identified as the most likely antigen recognized by NEO-201. **(B)** Flow cytometry analysis of the tumor cell lines ASPC-1, BxPC-3, CFPAC-1, and LS174T was performed to discriminate the native form of CEACAM5 and CEACAM6 from the NEO-201-reactive variant forms of CEACAM5 and CEACAM6. NEO-201 binding on cancer cell line model was assessed using NEO-201 Pacific Blue conjugated antibody. **(C)** NEO-201 binding to different forms of CEACAMs, other than 5 and 6, was measured by ELISA. **(D,F)** To confirm the binding of NEO-201 to CEACAM-5 and−6, CEACAM-negative cells HEK293T were transfected with CEACAM-5 and 6 siRNA. The two proteins, which express a different molecular weight, were equally bound by NEO-201. As proof of concept, CEACAMs and NEO-201-positive cells LS174T were knock-down for CEACAM 5/6 genes, which resulted in an about 25% partial loss of NEO-201 binding when a single gene was knock-down, and a further decreased to about the 50% loss of NEO-201 binding by the combined knockdown of both CEACAMs. **(E)** Western blot confirmed that NEO-201 binds an antigen with two different molecular weights in cell line models.

To identify the specific antigen recognized by NEO-201, protein lysates from OV90 and CFPAC1 were immunoprecipitated with 1 μg/ml or 10 ng/ml of NEO-201 in the presence of protein A beads and run on an acrylamide gel. Beads alone were used as negative control. The blot was probed with NEO-201 to confirm the selective isolation of the protein bound by the NEO-201. NEO-201 1 μg showed the best results in terms of protein immunoprecipitation for the proteomic analysis, while 10 ng/ml was not considered sufficient to achieve an adequate result (Figure 2A). OVCAR8 cells were used as negative control since they do not express the antigen recognized by NEO-201 (Figure 2A). Immunoprecipitated proteins were analyzed by mass spectrometry analysis to identify the antigen recognized by NEO-201. A list of possible antigens was detected comparing the proteins identified in the OV90, CFPAC1, OVCAR8, and protein A beads. Non-specific peptides were eliminated by subtracting those found in the negative control cell line OVCAR8 or in IgG control precipitates, and only the proteins detected in both OV90 and CFPAC1 were considered as relevant. From these screening, the carcinoembryonic antigen-related cell adhesion molecule (CEACAM)5, also known as CEA, and CEACAM 6 were identified as the most likely targets of NEO-201 (Figure 2A, bottom).

Dual staining of ASPC-1, BxPC3, CFPAC-1, and LS174T cell lines was performed with NEO-201 and with either anti-CEACAM5 or anti-CEACAM6 antibodies and analyzed by flow cytometry, showing that there is overlap between CEACAM5 or 6 expression with the antigen recognized by NEO-201 (Figure 2B). In most cell lines, however, the overlap was incomplete, suggesting that the cells express a normal variant of each CEACAM as well as the cancer-associated variant. By ELISA, NEO-201 antibody bound to both recombinant human CEACAM5 and CEACAM6 but not CEACAM1 or CEACAM8 (Figure 2C).

We proceeded to overexpress CEACAM5 and CEACAM6 in HEK293T cells to determine which reacted to NEO-201. HEK293T cell lines are known to have a negative phenotype for NEO-201 binding and do not express CEACAM 5 or 6 at baseline. HEK293T transfected with an empty vector confirmed no expression of either CEACAM5, or CEACAM6, or reaction to NEO-201 immunoblot, and the transfected clones showed a positive expression for either CEACAM 5 or CEACAM6, as intended. By Western blot, NEO-201 reacted with both CEACAM proteins (Figure 2D).

Evaluation of the expression of the antigen recognized by NEO-201 in ovarian and colon cancer cell lines by Western blot showed that LS174T colorectal cancer cell line expresses two distinct molecular weights of the antigen recognized by NEO-201, likely representing both CEACAM5 and CEACAM6, consistent with the flow cytometric analysis (Figure 2E). Among the ovarian cancer cells, PEO-1 and OV90 expressed only the lower CEACAM6 molecular weight form. SW480, OVCAR5, OVCAR8, and PEO4 were negative. Knockdown of either CEACAM5 or CEACAM6 in LS174T cells showed about 25% partial loss of NEO-201 binding, which was doubled to approximately 50% loss of NEO-201 binding by the combined knockdown of both CEACAMs (Figure 2F).

Altogether, these results confirmed that NEO-201 binds different types of cancers. The binding is highly selective for the tumor cells without cross reactivity with the surrounding healthy tissue. Moreover, within cancer tissue origins, the antigen recognized by NEO-201 is differentially expressed between tumor histological and/or molecular subtype. These data suggested that NEO-201-positive tumors express a specific phenotype of a tumor-associated variant of CEACAM 5 (CEA) and 6, which is not expressed in normal tissues.

### Mutational Analysis

To investigate the nature of CEACAM 5 (CEA) and 6 variants expressed on tumor cells, we performed whole exome sequencing of OV90 (NEO-201^pos^), LS174T (NEO-201^pos^), SW480 (NEO-201^neg^), and OVCAR8 (NEO-201^neg^) cell lines. We searched for mutations that were commonly present in both LS174T and OV90 (NEO-201-positive cell lines) but not in SW480 and OVCAR8 (NEO-201 negative). Interestingly, gene analysis failed to show any mutations in the CEACAM family genes. Instead, missense mutations of the zinc-finger protein ZNF141 and major histocompatibility complex HLA-DRB5 genes were detected in both OV90 and LS174T and not in the OVCAR8 or SW480 cell line. Although the role of ZNF141 in cancer is not clear, other zinc finger proteins are known to bind either DNA or RNA and to play a role in gene expression, post-transcriptional modification, and protein trafficking, and may correlate with metastatic process and EMT transformation. HLA-DRB5 is a key component in the antigen presentation process. Mutations in this gene could mediate cancer cell immune escape, but it is currently unclear how it may relate to the expression of the antigen recognized by NEO-201.

### NEO-201 Alone Does Not Affect Tumor Cell Viability

To determine the biological significance of NEO-201 reactivity with cell lines, we investigated its effect on viability of OV90 and LS174T *in vitro*. OV90 cells exposed to increasing concentrations of NEO-201 for 72 h showed no change in viability (data not shown).

### NEO-201 Mediates ADCC Killing Against Human Ovarian and Colorectal Carcinoma Cell Lines *in vitro*

**Figure 3:**
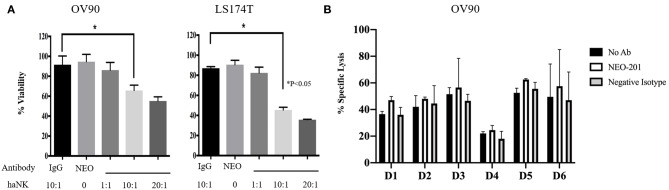
NEO-201 decreases cell viability by activating natural killer (NK)-mediated antibody-dependent cellular cytotoxicity (ADCC) *in vitro*. **(A)** NEO-201-positive OV90 and LS174T cells lines were selected to assess the effect of NEO-201 on NK-mediated ADCC. Cells were incubated with either highly active natural killer (haNK), NEO-201 at a dose of 1 μg/ml, or a combination of NEO-201 with haNK at different effector-to-target (E:T) ratios. ADCC activity was assessed by non-radioactive ADCC assay. Experiment and results from **(A)** were analyzed according to the Celigo program manufacture as described in the Material and Methods section. **(B)** In order to select the best NK human donor to use for *in vivo* experiments, the OV90 cell line was incubated with the NK isolated from the PBMCs from six different donors in the presence of 1 μg/ml of NEO-201. To evaluate specific lysis, NK derived from a selected human donor were activated with IL-2 and used for the experiment in two different effector cells (E):target cells (T) ratios: 50:1 or 100:1. Chromium released assay was used to evaluated specific lysis. Experiments depicted in this figure reflect the mean ± SD of three independent experiments. Ordinary one-way ANOVA was used for statistical analysis. *P < 0.05.

To evaluate the ability of NEO-201 to kill tumor cells through NK-mediated ADCC, OV90 or LS174T cells were incubated with either IgG isotype control or NEO-201 at 1 μg/ml, with/without the highly active NK cell line (haNK) for 4 h. Neither NEO-201 nor haNK +IgG isotope control showed any significant effect on cell viability (Figure 3A). We evaluated the effect of NEO-201 on NK-mediated ADCC on OV90 and LST174T using effector-to-target (E:T) ratio of 1:1, 10:1, and 20:1. The combination of NEO-201 with haNK showed a statistically significant increase in ADCC in both cell lines at the ratio of 10:1 and 20:1. No significant effect on ADCC was observed at the ratio of 1:1. These data indicate that NEO-201 does not have a direct cytotoxic effect on the tumor cells, suggesting that the NEO-201 targets, CEACAM 5 and 6, have no role in cell proliferation. Instead, these data strongly suggest that the anti-tumor activity of NEO-201 is mediated by the activation of ADCC in both ovarian and colon cancer cells. This is consistent with our previous results confirming the role of NEO-201 in triggering NK-mediated ADCC using anti-CD16 antibody to block NEO-201-induced ADCC (13).

We next screened NK from different donors in order to further optimize the pre-clinical model. A ^51^Chromium release ADCC assay guided the selection of the NK donor to be used for the animal study (Figure 3B). Based on these results, D3 NK was selected to be used in the animal study.

### NEO-201 Efficacy in Ovarian Cancer Orthotopic Tumor Model *in vivo*

**Figure 4:**
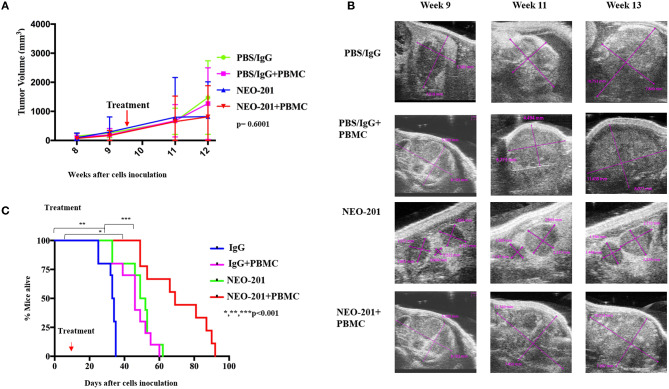
Antiproliferative response to NEO-201 and survival in model of ovarian cancer *in vivo*. **(A)** OV90 cells were injected in the mice ovary bursa and observed for their ability to develop tumors. Tumors were allowed to grow until they reached at least 100 mm^3^, about 8 weeks post cell injection. Tumor size was followed by weekly ultrasound. After randomization, each mouse received one cycle of treatment, which consists of two doses of drug on day 1 and day 7 of week 10. Curves represent tumor volume when treated ×3 with either control {phosphate-buffered saline (PBS)/IgG}, PBMCs (8 million cultured in 200 U/ml IL2 overnight), NEO-201 (250 μg), or their combinations. Kruskal–Wallis test was used to evaluate significance (*p* = 0.6001). **(B)** Representative ultrasound images of tumor-bearing mouse ovaries. Purple lines indicate cross sectional measurements from which volume was calculated. **(C)** OV90 cells were injected in the mice peritoneum to reproduce an orthotopic model of peritoneal carcinomatosis from ovarian cancer spreading. Tumors were allowed to grow for 2 weeks before being randomized in the four groups of treatment as described above. Curves represent survival. Significance was evaluated by Mantel–Cox. *P < 0.05, **P < 0.01, ***P < 0.001.

We designed our mouse model to incorporate the ADCC mechanism of tumor cell killing. Because the NEO-201 antibody is specific to human protein, we are unable to use an immune-competent mouse model of ovarian cancer. We therefore used human xenografts in nude mice and inoculated IL-2-activated human PBMCs at the time of NEO-201 injection. For the first model, mimicking primary ovarian cancer, we inoculated OV90 cells into the bursal sac surrounding the mouse ovary in order to initiate a local orthotopic primary tumor that could be measured over time using ultrasound imaging. In the ovarian bursa model (Figures 4A,B), the treatment with NEO-201 or NEO-201 in combination with PBMCs, showed a trend toward tumor control. In a second model, we inoculated the OV90 cells into the peritoneal cavity in order to mimic disseminated ovarian cancer and peritoneal carcinomatosis. We used this model to measure the effect of NEO-201 on overall survival. Mice that received NEO-201 with activated PBMCs experienced the longest survival (Figure 4C) (*p* < 0.0001). In this model, NEO-201 alone is also able to improve survival compared to the isotype control treatment. This could be due to direct anti-tumor effects of the antibody in this setting or to the activation of the mouse innate immune system. PBMCs alone had a similar partial effect, likely due to allo-reactivity of the immune cells against a tumor with a different MHC haplotype. The combination of NEO-201 and PBMCs dramatically improved survival over vehicle or either treatment alone. Overall, these mouse models demonstrate *in vivo* activity of NEO-201 against ovarian cancer that specifically expresses the antibody target.

## Discussion

Recent efforts in cancer therapeutics have focused on the development of drugs that activate the immune system against cancer cells to achieve durable disease control with a physiological strategy and avoiding chemotherapy side effects. The efficacy of immunotherapy has been limited to specific tumor types and correlated with tumor genetic instability due to deficiency of mismatch repair (MMR) and/or the expression of PD-L1 (24). Therefore, there is urgent need for alternative therapeutic approaches to improve immunotherapy efficacy against other cancers. The innate immune system, including monocytes and NK cells, likely works in conjunction with adaptive immunity, to support and sustain anti-tumor activity. Here, we assessed a novel monoclonal antibody for its ability to direct ADCC activity of NK cells against cancer cells expressing an apparent tumor-associated variant of CEACAM-5 and CEACAM-6.

The CEACAMs are a group of cell surface glycoproteins, which are normally expressed on the surface of the epithelial cells, where they regulate tissue architecture and signal transduction (25). They are overexpressed in several tumors where they have been linked to cell migration and metastatic process, and drug resistance (25, 26). Moreover, post-translational modifications occurring during tumorigenesis, such as glycosylation, could result in a different composition of the glycan groups expressed by the CEACAMs (27–29). Colorectal cancers may demonstrate increased expression of mannose, Thomsen–Friendenreich antigen, and sialylation compared to healthy colon tissue (30). Similarly, fucose and mannose can be increased, while N-acetylgalactosamine, N-acetylglucosamine, galactose, branched and bisecting N-glycansin may be lower than normal (31). These modifications could alter cell-to-cell and cell-to-extracellular matrix (ECM) intercellular interaction (32). It is possible that a difference in glycosylation pattern explains the specific binding of NEO-201 to specific tumor-associated CEACAM-5 and CEACAM-6 variants but not to those expressed on healthy tissues as shown by the immunohistochemistry analysis. Interestingly, we also observed that carcinoma cell lines, expressing native forms of CEACAM-5 and CEACAM-6, showed a different profile for expression of the antigen recognized by NEO-201. This data supports the hypothesis that the antigen recognized by NEO-201 is a specific tumor-associated variant of CEACAM-5 and -6.

Mutational analysis was conducted, but no CEACAM gene alteration was found in the cell lines, which had a positive reactivity with NEO-201. Instead, a gene mutation of zinc-finger protein ZNF141 was found (33). The zinc-fingers are a group of protein, which was initially identified as transcription factors, and recent studies showed that this group of protein could be involved in multiple cellular processes other than gene expression (34). Although ZNF141 expression/role in cancer has not yet been clarified (35), overall zinger-finger proteins have been associated with tissue development abnormalities and epithelial–mesenchymal transformation (36). ZNF141 mutations could be involved into a regulation of the different glycosylation status of CEACAM 5 and 6 that are targeted by NEO-201; however, further studies are needed to better understand the presence and relevance of this mutation in human cancers.

Although NEO-201 showed no direct cytotoxic effect as other mAbs like Trastuzumab (37), Cetuximab (38), or Rituximab (8), it can exert a significant anti-tumor activity not only inducing NK-mediated ADCC but also enhancing direct NK killing against tumor cells. Recently, we showed that NEO-201 mediated enhancement of NK killing against CEACAM5^+^/NEO-201^+^ human carcinoma cell lines, demonstrating that the binding between NEO-201 and the tumor variant of CEACAM5 can block the interaction between CEACAM5 on tumor cells and CEACAM1 on NK cells to reverse CEACAM1-dependent inhibition of NK cytotoxicity (39). Similarly, another study demonstrated the ability of an anti-CEACAM5 monoclonal antibody (CC4) to restore NK cytotoxicity in colorectal cancer preclinical models by blocking the CEACAM 5 (CEA)–CEACAM 1 axis (40).

In addition, as previously reported by our group, NEO-201 has antitumor activity *in vitro and vivo*. Our previous work demonstrated that NEO-201 induced cancer cell killing through activation of CDC and ADCC in pancreatic cancer models. *In vivo* NEO-201 reduced the growth of human pancreatic tumor xenografts in mice and demonstrated safety/tolerability in non-human primates with a transient neutropenia lasting ~8 days as the only adverse effect observed (10). Furthermore, in another study, we have also proved that the stimulation of NK cells with IL-15 superagonist further enhanced the NEO-201-mediated ADCC against cell lines expressing the antibody target *in vitro* (13). Here, we demonstrate the *in vivo* antitumor activity of NEO-201 in a preclinical model of ovarian cancer. The treatment with NEO-201 plus PBMCs dramatically improved survival of mice compared to vehicle or either treatment alone (Figure 4), suggesting an *in vivo* activity of NEO-201 against ovarian cancer that specifically expresses the antibody target.

All together, these data suggested that NEO-201 has an antitumor activity and safety profile that we moved forward to clinical validation in a first in human clinical trial that is now ongoing at the NCI (NCT03476681). Targeting an antigen expressed in tumors, but not in normal tissues, allows patient selection for optimal treatment. The antigen recognized by NEO-201, a variant of CEACAM-5 and CEACAM-6, is specific to cancer tissue but expressed across cancer subtypes. Interestingly, it was developed originally using colon cancer tissue, and appears to be expressed predominantly in tumors of gastrointestinal origin or mucinous phenotype. NEO-201, therefore, has both therapeutic and diagnostic potential. Future studies will incorporate companion diagnostics during the course of clinical development in order to identify patient populations who express the antigen recognized by NEO-201 and are most likely to benefit from this potential therapeutic agent targeting tumor-specific variants of CEACAM-5 and CEACAM-6.

## Data Availability Statement

The original contributions presented in the study are publicly available. This data can be found here: the NCBI BioProject, ID 625511 (https://www.ncbi.nlm.nih.gov/bioproject/625511).

## Ethics Statement

The studies involving human participants were reviewed and approved by protocol 99-CC-0168, approved by the Institutional Review Board of the National Cancer Institute. The patients/participants provided their written informed consent to participate in this study. The animal study was reviewed and approved by NCI Animal Care and Use Committee.

## Author Contributions

MM wrote the manuscript and analyzed the data. KZ, JD, MN, LH, MO, DA, SH, AO-T, TA, SD, and MF performed the experiments and analyzed the data. JL and AA analyzed the data. PA, KT, and CA designed the experiments, interpreted the data, edited the manuscript and provided administrative, technical, and material support.

## Conflict of Interest

JD, MF, KT, and PA conducted this research as employees of Precision Biologics, Inc. PA has ownership interest in Precision Biologics, Inc. CA received research funding from Precision Biologics. Precision Biologics had a role in the study design, data collection and analysis of the *in vitro* experiments; and had a role in the preparation of the manuscript. The remaining authors declare that the research was conducted in the absence of any commercial or financial relationships that could be construed as a potential conflict of interest.
